# Comparing neural models for nested and overlapping biomedical event detection

**DOI:** 10.1186/s12859-022-04746-3

**Published:** 2022-06-02

**Authors:** Kurt Espinosa, Panagiotis Georgiadis, Fenia Christopoulou, Meizhi Ju, Makoto Miwa, Sophia Ananiadou

**Affiliations:** 1 National Centre for Text Mining, Department of Computer Science, The University of Manchester, Manchester, UK; 2 Department of Computer Science, University of the Philippines Cebu, Gorordo Avenue, Lahug, Cebu City, Philippines; 3 Alan Turing Institute, London, UK; 4 Toyota Technological Institute, 2-12-1 Hisakata, Tempaku-ku, Nagoya, 468-8511 Japan; 5 Artificial Intelligence Research Centre (AIRC), National Institute for Advanced Industrial Science and Technology (AIST), 2-3-26 Aomi, Koto-ku, Tokyo, 135-0064 Japan

**Keywords:** Event extraction, Biomedical text, Nested events

## Abstract

**Background:**

Nested and overlapping events are particularly frequent and informative structures in biomedical event extraction. However, state-of-the-art neural models either neglect those structures during learning or use syntactic features and external tools to detect them. To overcome these limitations, this paper presents and compares two neural models: a novel EXhaustive Neural Network (EXNN) and a Search-Based Neural Network (SBNN) for detection of nested and overlapping events.

**Results:**

We evaluate the proposed models as an event detection component in isolation and within a pipeline setting. Evaluation in several annotated biomedical event extraction datasets shows that both EXNN and SBNN achieve higher performance in detecting nested and overlapping events, compared to the state-of-the-art model Turku Event Extraction System (TEES).

**Conclusions:**

The experimental results reveal that both EXNN and SBNN are effective for biomedical event extraction. Furthermore, results on a pipeline setting indicate that our models improve detection of events compared to models that use either gold or predicted named entities.

## Background

Understanding the functionality of biological systems requires knowledge of the complex associations across multiple levels of biological organisation [1]. Complex associations such as molecular events could be responsible for drug reactions or development of certain diseases [2]. Until recently, efforts in Information Extraction were primarily focused on recognising mentions of relevant entities such as genes and proteins [3] or on the extraction of pairwise relations such as drug-disease relations, drug-drug [4] and protein-protein interactions [5]. Since these binary relations are too restrictive and cannot capture the complexity of associations between biological elements [6], there has been increasing interest in Information Extraction approaches for the extraction of structured representations, capable of capturing associations between an arbitrary number of elements [7]. *Event extraction* provides a way to represent structured information from unstructured text. More specifically, in the biomedical domain, an event refers to the change of state of one or more biomolecules (eg. genes and proteins) or interactions between them, and is represented by a trigger, usually a verb or its nominalised form, and a set of unordered arguments, usually entities, with their corresponding roles (i.e. relations) to the trigger [8] . Such representations can be useful in information retrieval and question answering systems, for creating biological networks or for inferring new associations [9].

For this purpose, several evaluation tasks, such as BioNLP’09 [10], BioNLP’11 [11] and BioNLP’13 [12] shared tasks, have been held to allow comparisons of advanced methods for biomedical event extraction. Event structures can be divided into three categories: *Flat events*, that correspond to structures where all arguments are named entities. *Nested events*, that consist of at least one argument which is an event and *overlapping events* that share at least one common argument.

In this work, we focus on nested and overlapping event structures. These structures occur widely in biomedical text and are particularly important since they can capture different relations between events. In contrast to relations between named entities, relations between events have a richer structure and thus are more useful in domains, such as Biomedicine, where relations are typically more complex.

**Fig. 1:**
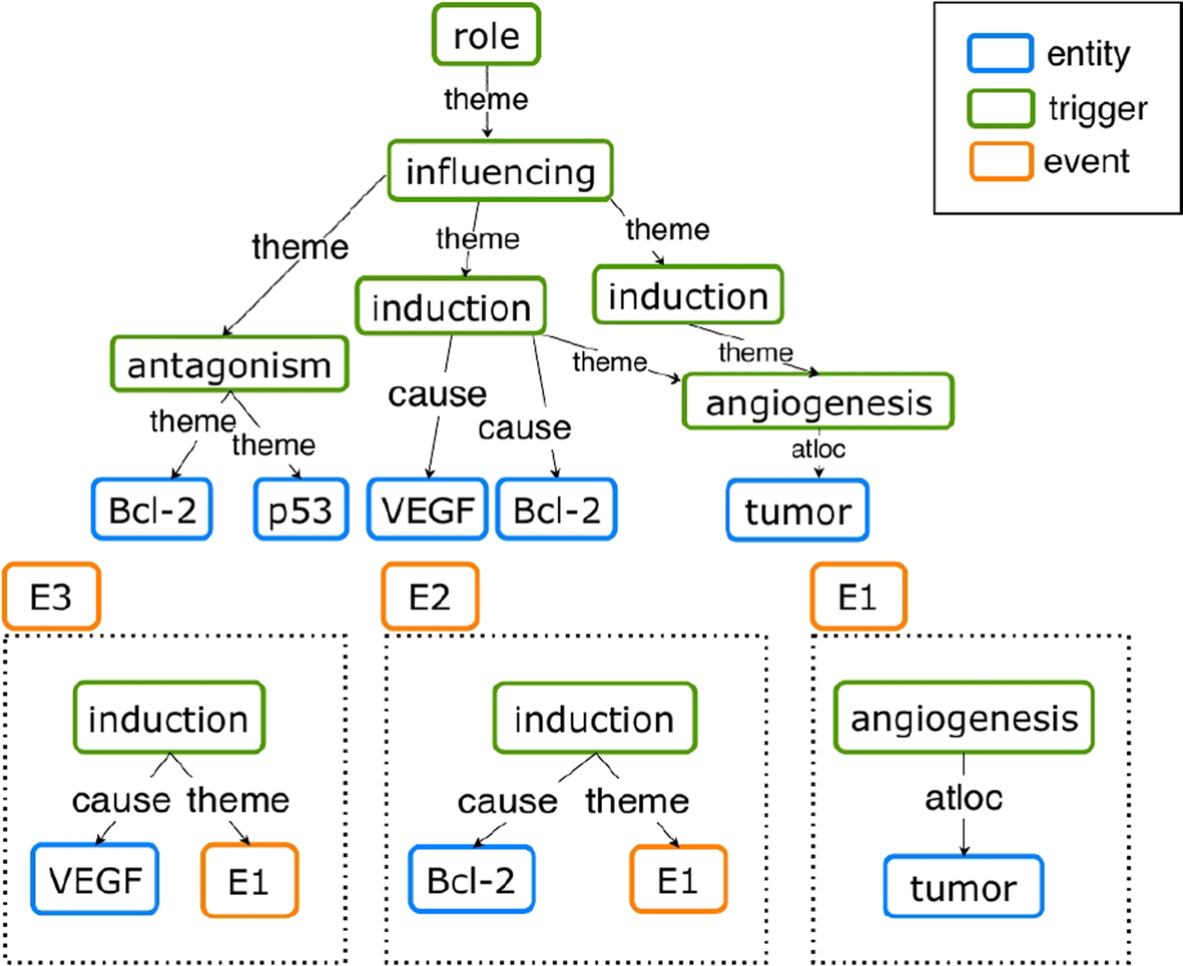
Top: A DAG-structured relation graph (topmost) from the sub-sentence “*We discuss the role of this transcription factor in influencing Bcl-2/VEGF induction of tumor angiogenesis, ...*” from BioNLP’13 CG Shared Task  [14]. Bottom: A pair of overlapping and nested events (*E*2, *E*3) extracted from the graph with their shared argument event, a flat event (*E*1)

Figure 1 illustrates an example sentence in the biomedical domain. For this sentence, a relation graph is constructed using triggers and entities as nodes and binary relations between them as edges. The figure exhibits a Directed Acyclic Graph (DAG) structure. Unlike a tree structure, DAGs allow multiple paths between two nodes and as such are more appropriate to represent event structures [13]. The DAG-structured relation graph (topmost) encapsulates 3 event structures in total. It contains the nested event structures [13] *E*2 and *E*3 and a flat event *E*1. Moreover, *E*2 and *E*3 are at the same time overlapping events^1^ (explicitly shown in the relation graph) because they share a common argument, *E*1.

### Related work

Most research on biomedical event extraction was advanced by the development of shared tasks from the BioNLP community. The initial shared task [10] was focused on bio-molecular events. As an extension, BioNLP Shared Task 2011 [11] introduced five tasks: the GENIA task (GE), which focuses on transcription factors in human blood cells, Epigenetics and Post-translational Modification (EPI), Infectious Diseases (ID), Bacteria Biotopes (BB) and Bacteria Interactions (BI). The most recent BioNLP Shared Task 2013 [12] proposed new tasks to handle events on cancer genetics and pathway curation.

In addition to the shared tasks, approaches for biomedical event extraction are predominantly pipeline systems [15] that decompose event extraction into a set of subtasks, as follows: (i) *trigger/entity detection*, that determines which words and phrases in a sentence potentially constitute participants of an event, (ii) *relation detection*, that finds pairwise relations between triggers and candidate arguments, (iii) *event detection*, that combines pairwise relations into complete event structures.

Joint learning approaches have been explored [16–19], with a focus on finding relation graphs and detecting events using rules. Unlike those approaches, McClosky et al. [13] modelled events into tree-structures using dependency parsing, thus ignoring overlapping events. Most recently, Trieu et al. [20] proposed an end-to-end nested event extraction model based on large language models. Zhu and Zheng [21] also developed a joint end-to-end event extraction model that uses a penalty based strategy to reconstruct nested events. Instead of a joint learning approach, we focus on the pipeline setting to gain a better understanding of the contribution of each component to the extraction of nested events.

Neural methods for event extraction were also explored in the newswire domain [22, 23]. They were mainly applied on the ACE 2005 dataset which does not contain nested events [24], and as a result cannot detect nested and overlapping events.

### Objectives

We compare two neural models for nested and overlapping event detection: (i) a novel EXhaustive Neural Network (EXNN) model, where all the possible event structures are generated from predicted relations and detected as an event or not, and (ii) a Search-based Neural Network (SBNN) model [25] that detects overlapping and nested events with beam search. For both models, we describe in detail the candidate events construction process, which is performed on binary relations to create DAG structures. On these structures we detect nested and overlapping events in a bottom-up manner. We compare our models against the event detection component of the state-of-the-art pipeline event extraction system Turku Event Extraction System (TEES) [15] and evaluate them on more datasets in BioNLP Shared Task 2013 than previous work [25]. Finally, we conduct in-depth analysis to determine the strengths and weaknesses of each model.

## Methods

### Neural models

#### Search-based model

**Fig. 2:**
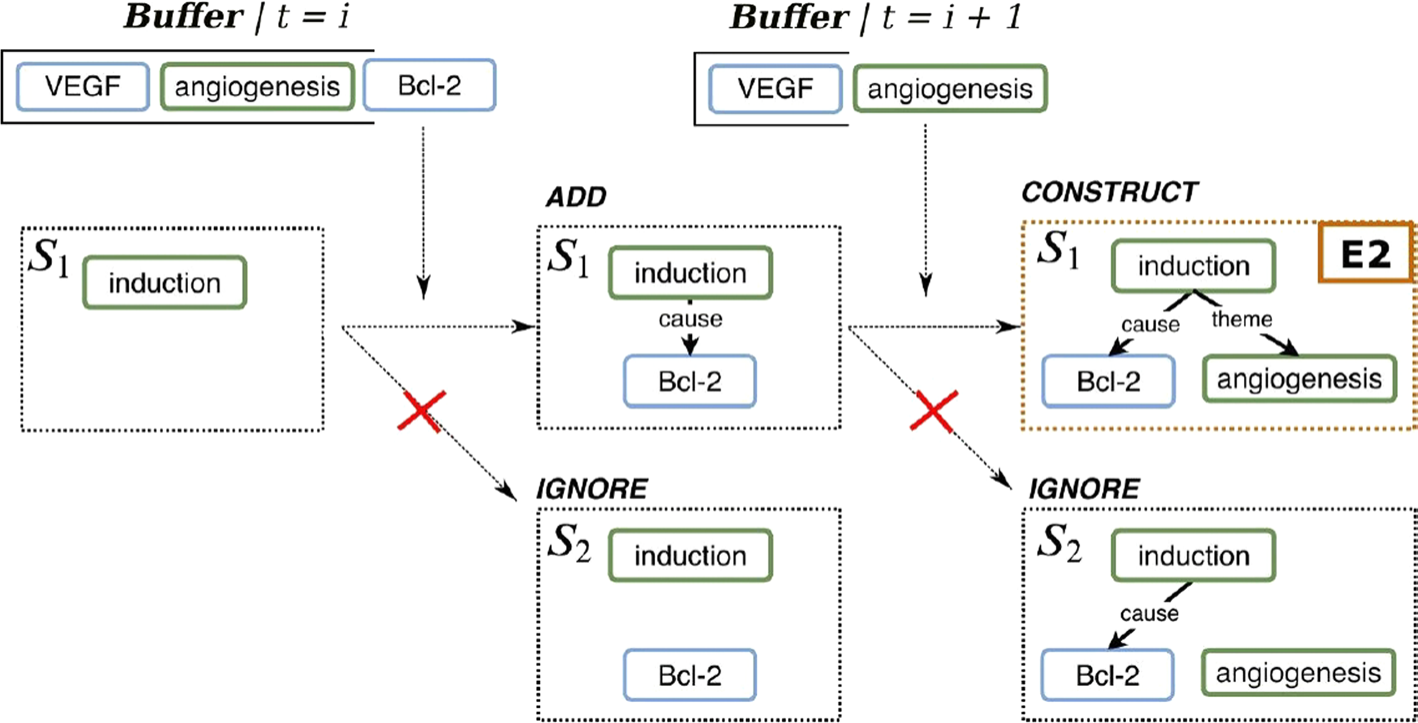
An example of the search procedure of the Search-based neural network (SBNN). After one ADD and one CONSTRUCT actions the model is able to detect *E*2. The *Buffer* contains the arguments of the previous level. Only one beam (k = 1) is expanded in this example for brevity

We evaluate the existing SBNN model [25] on more event datasets than already reported. Moreover, we investigate the upper- and lower-bound performances of the model in a pipeline setting trained on multiple scenarios. In detail, SBNN constructs events from a relation graph by structured prediction. It resembles an incremental transition-based parser [26] that considers the search order, actions and representations composed in DAG structures. Transition-based methods have been investigated only for flat structures so far [27, 28], therefore, to address the overlapping and nested structures, our model performs beam search on relation graphs to select actions for event construction. We define three actions applied at each time step to each event argument: add the argument (ADD), ignore the argument (IGNORE) and add the argument and construct an event candidate (CONSTRUCT). We use *all* the beams instead of the the best path only [26] to enable prediction of overlapping and nested events. Figure 2 shows a snapshot of the search procedure within one time step as applied to a relation graph to detect event *E*2 (see Fig. 1). SBNN is parameterized by a value k that controls the width of beam search. A high value of k allows multiple paths to be expanded, but increases the computational complexity of the model.

Candidate event structures are generated using search and then the predicted events are constructed for each trigger of the relation graph in a bottom-up manner. The model predicts flat events first and then the representations of the flat events become the arguments of the nested events. The search process terminates when no flat events are detected (see Additional file 1: Schematic architecture).

#### Exhaustive model

**Fig. 3:**
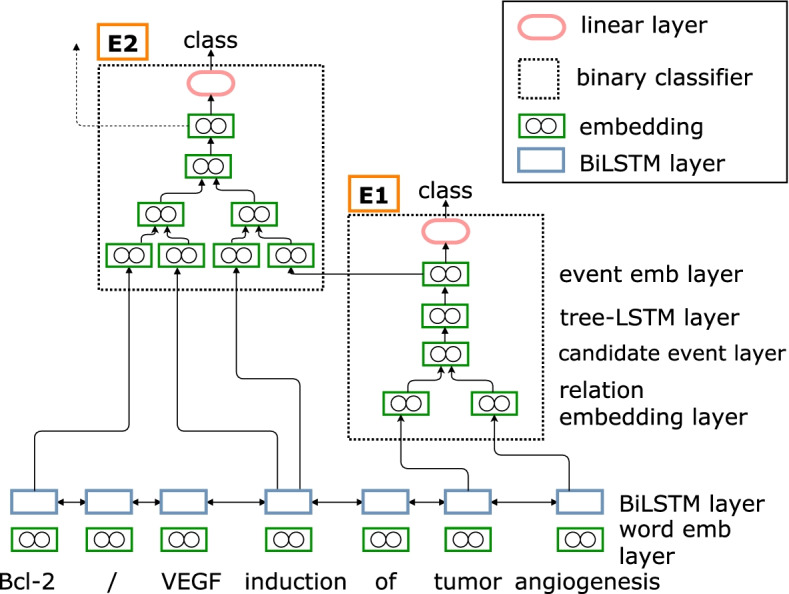
A schematic of the exhaustive neural networks (EXNN) architecture during the bottom-up detection of events *E*1 and *E*2 of Fig. 1

In contrast to SBNN, the exhaustive approach generates all the possible candidate event structures from a given set of relations instead of performing a search over them. In detail, we develop a tree-LSTM based model, which differentiates it from the exhaustive approach in [20]. Our model input consists of the candidate structures associated with each trigger. For each sentence, we prepare contextualised word representations using a Bidirectional Long-Short Term Memory (BiLSTM) network [29] which is shared among all event structures. Similar to the SBNN model, we represent each relation by concatenating the representation of the trigger, the role and the argument for each event structure. We use the relation representation without actions as its input. Each relation representation is also concatenated with *IN* or *OUT* embeddings, which are trainable parameters, to indicate if the relation is part (*IN*) or not part (*OUT*) of the event structure. We concatenate the multiple relation representations of an event structure (i.e., candidate event layer in Fig. 3).

Since the EXNN does not consider actions and search orders, including the order of arguments, we employ a Child-Sum Tree-LSTM [30] on the concatenated relation representations. The Child-Sum Tree-LSTM allows the network to selectively incorporate information from each child, i.e., each relation of each candidate event structure. We then create an event representation from the output of the Tree-LSTM. This representation is passed through a multi-layer perceptron with a non-linear activation to produce a reduced representation of 2 values, representing the number of classes (event or no event). Finally, using a softmax activation function the structures are classified as forming an event or not.

### Candidate events construction

**Fig. 4:**
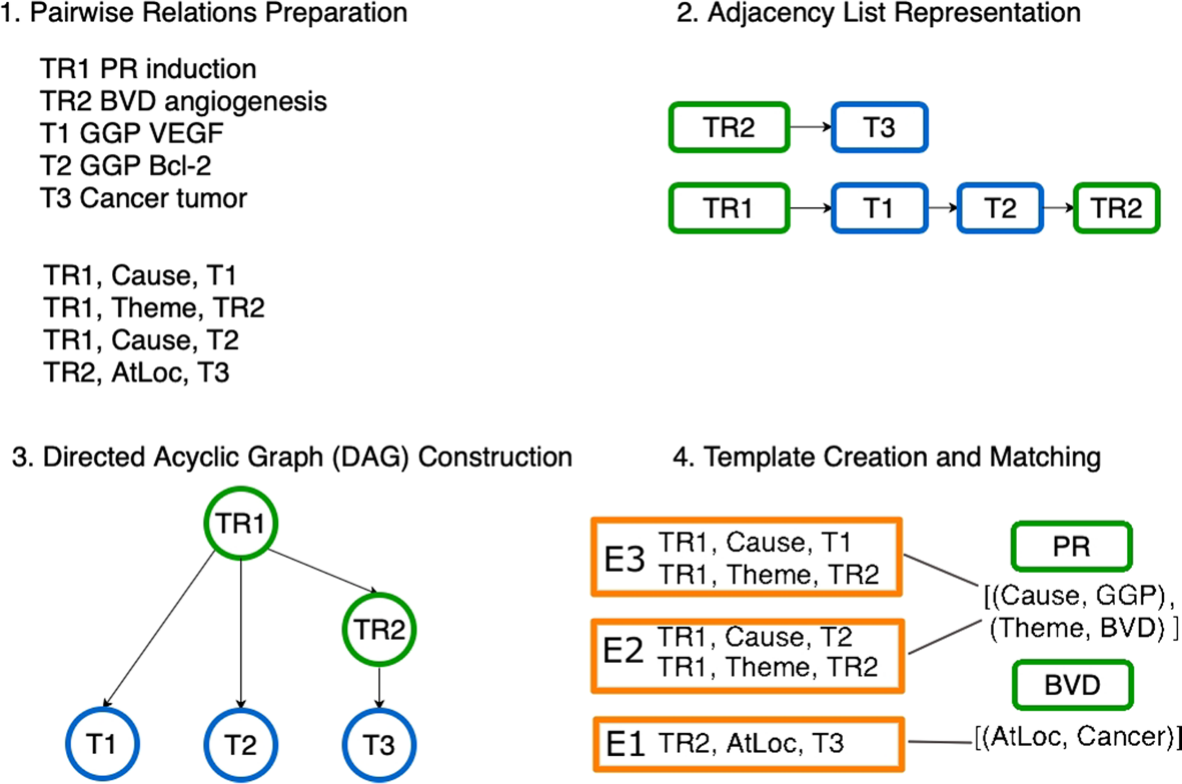
An illustration of the candidate events construction process given pairwise relations extracted from the relation graph in Fig. 1 involving events *E*1, *E*2 and *E*3. Nodes in green correspond to triggers and nodes in blue correspond to entities

As shown in Fig. 4, we construct candidate events based on pairwise relations in four steps. For our models, we assume that pairwise relations between triggers and arguments are given for each sentence.

The first step involves the preparation of the input relations. To construct these relations we break apart each event structure into all possible pairwise relations. A pairwise relation can be seen as a triple (*trigger, role, trigger/entity*). In Step 1 of Fig. 4, we show the IDs of triggers (starting with TR) and entities (starting with T), respectively. For instance, the first relation triple contains: *TR1* which represents *Positive Regulation* (PR) as the trigger, **Cause** as the role, and *T1* which represents *Gene-or-Gene-Product* (GGP) as the argument. In the second relation triple, the argument *TR2* which represents *Blood Vessel Development* (BVD) is a trigger itself and as such this is an example of a nested event structure.

The second step involves the creation of an adjacency list for each trigger. In detail, we create a list for each trigger and add all the arguments that are associated with it. For example, the list associated with the *TR1* trigger has three arguments, while the list for *TR2* contains only one argument.

The third step is the construction of the DAG structure. Triggers or entities represent nodes, while pairwise relations correspond to directed edges between two nodes, either between two triggers or a trigger and an entity. Following [31, 32], we create a topological sorting of the given trigger-argument relations. A topological sort or topological ordering of a directed graph is a linear ordering of its vertices such that for every directed edge *uv* from vertex *u* to vertex *v*, *u* comes before *v* in the ordering. In the resulting DAG structure shown in Step 3, some arguments are triggers such as *TR2*.

The last step involves the template extraction and matching process. We build event structure patterns from the training data and use them as templates of valid event structures. The uniqueness of event structures is based on a multiset representation, since an event argument (Role, Argument Type) can appear multiple times in an event structure. For example, for events *E*1, *E*2 and *E*3 in Fig. 1, we create the respective multiset representation under PR and BVD as shown in Step 4. We extract templates for each event type $t \in T$ resulting in a set of templates $F_t$. To perform template matching, we create a multiset (*m*) representation $A_t^{(m)}$ for each candidate event structure *A* of type *t*. Then, we compare the multiset representation $A_t^{(m)}$ to each $F_{t}$ representation in the templates of type *t*. If $A_t^{(m)}$ does not correspond to any of the $F_{t}$ structures, then it is discarded, otherwise, it is considered a valid event structure.

### Bottom-up event structure classification

In this section, we describe the event detection process. We detect events from the bottom level of the DAG structure going up. This way, the representations of events in the lower level are used as arguments in the events of the upper levels.
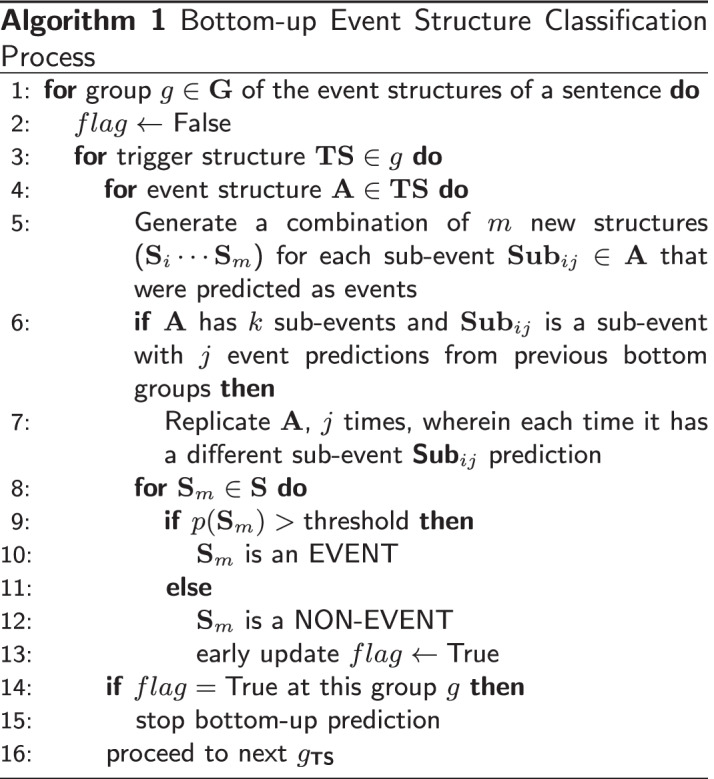


Algorithm 1 describes the procedure for bottom-up event structure classification. We apply the algorithm to the output of the candidate generation process described. We start event prediction at the bottom of the DAG structure, with no groups ($g=0$) in Line 1. Since this is a DAG structure, each group *g* can contain many trigger-structure pairs *TS*. In turn, each *TS* pair can contain many event structures *A*. This is the case where many events are associated with the same trigger and we refer to those as overlapping events.

**Fig. 5:**
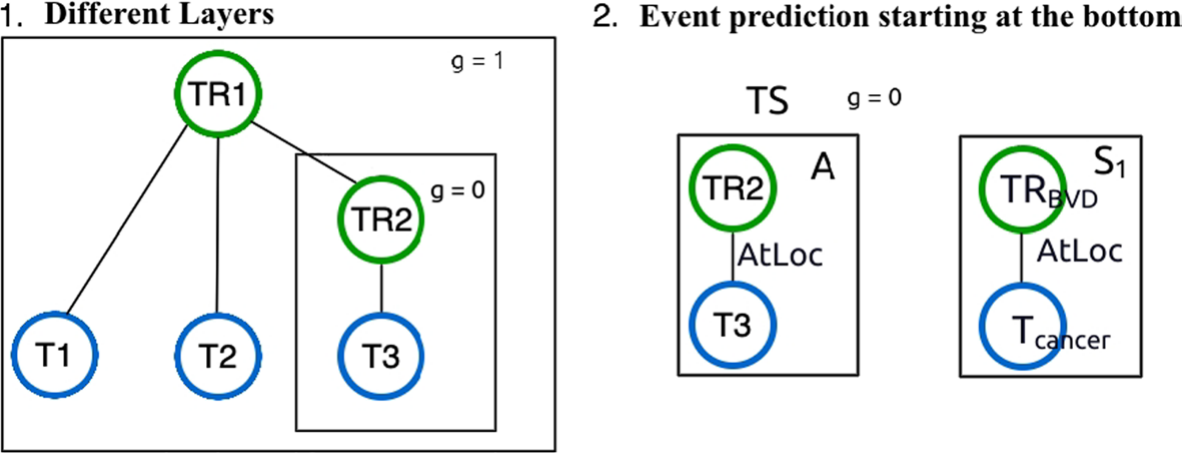
An example of the Algorithm 1 applied to the events of Fig. 4. At $g=0$ we see the detection of *E*1

Then for each event structure *A*, we check if any of its arguments are events. If there is an event argument which is a trigger, essentially representing another event, then we check how many events have been predicted previously for this trigger. We then generate as many new structures equivalent to the number of event predictions for that trigger or event argument, as stated in Lines 5–7. In effect, the event structures at the higher levels of the DAG structure use the event representations of the predicted event structures at the lower levels. For example, in Fig. 1, if we are currently predicting events under the trigger *influencing*, we check if any of its *n* arguments are events. If at a particular point in our algorithm, we consider both triggers (represented by *induction* and *angiogenesis*) as arguments, we check how many events have been predicted for each of these triggers. Since in this case there is only one event associated with *angiogenesis*, which is *E*1, only one structure is generated for the *angiogenesis* event argument, as shown in Fig. 5. On the contrary, if the trigger *induction* has *N* event predictions, then *influencing* has to be replicated *N* times since each sub-event prediction is unique. In Line 7 of the algorithm, we do this for each event argument of a prospective event structure *A*.

For each generated structure $S_m$, we score it using a neural network (Line 9) (see Additional file 2). This part is slightly different in the search-based model, since what will be scored by the neural network is the partially-built event structure. If the prediction score for the current event structure is below a specified threshold, the prediction process stops, otherwise, the process continues to the last group *g* of the event structures in the sentence (Lines 14–16).

## Results

### Evaluation corpora

We follow Björne and Salakoski [15] and evaluate our models on the following tasks: Cancer Genetics (CG), Pathway Curation (PC) and GENIA Event Extraction for NFkB knowledge base (GE) [12].

**Table 1:** An overview of the three BioNLP 2013 ST datasets

Dataset	Documents	Entity types	Event types
CG 2013	600 Abstracts	18	40
PC 2013	525 Abstracts	4	23
GE 2013	34 Full papers	2	13

**Table 2:** Details of the three BioNLP 2013 ST datasets

Dataset	Item	Train	Dev.	Test
CG 2013	Documents	300	100	200
	Sentences	2640	850	1610
	Pct unknown words	0%	10.63%	10.68%
	Events	9422	3217	5530
	Flat events	45.31%	44.07%	NA
	Nested events	34.95%	36.46%	NA
	Overlapping events	41.05%	43.05%	NA
	Inter-sentence events	4.08%	3.11%	NA
PC 2013	Documents	260	90	175
	Sentences	1900	660	1254
	Pct unknown words	0%	11.66%	11.50%
	Events	6,657	2320	4004
	Flat events	33.28%	34.74%	NA
	Nested events	38.90%	38.87%	NA
	Overlapping events	54.88%	52.80%	NA
	Inter-sentence events	4.70%	2.41%	NA
GE 2013	Documents	10	10	14
	Sentences	1051	1104	1188
	Pct unknown words	0 %	16.84%	17.07%
	Events	2882	3259	3301
	Flat events	53.57%	42.25%	NA
	Nested events	31.15%	38.96%	NA
	Overlapping events	26.57%	35.80%	NA
	Inter-sentence events	10.55%	22.55%	NA

Tables 1 and 2 illustrate the overview and detailed statistics of the BioNLP 2013 Shared Task datasets. The total percentage of the breakdown of events does not equal to $100\%$ because nested and overlapping events may have an intersection, meaning, a nested event can be an overlapping event and vice versa. The CG dataset contains the largest number of entity and event types, annotated events, documents and sentences. We can observe that while the CG and PC datasets have a relatively low number of inter-sentence events, the GE dataset includes a higher number of inter-sentence events. Furthermore, the GE dataset was constructed using full papers while the other two datasets include only abstracts which contain very condensed and summarised information. This results in the relatively higher ratio of unknown (UNK) words (i.e., words not seen in the training set) in the GE dataset compared to the PC and CG datasets. Note that the percentage of unknown words in the dev/test set is computed with respect to the vocabulary of the training set in each dataset (i.e. the percentage of unknown words in the training set is zero).

In this work, we focus only on sentence-level events. The information of flat, nested and overlapping events is not available for the test sets since the annotations for the test sets are not provided and instead the evaluation is performed by uploading the predictions to the task organizers servers [15].

We use the official evaluation script [12] to measure the performance of the model on nested, overlapping and flat events, which uses approximate span and recursive event matching. We first separate the nested, overlapping and flat events, respectively. Then we compute Precision (P) and Recall (R) for each category and in particular for nested events, we compute them as follows:

$$\begin{aligned}& \text {Precision}= \frac{\# \,\text {Correctly predicted nested events}}{\# \,\text {All predicted nested events}}, \\& \text {Recall}= \frac{\# \,\text {Correctly predicted gold nested events}}{\# \,\text {All gold nested events}} \end{aligned}$$

The evaluation script detects nested events by comparing the whole tree structure down to its sub-events until it reaches the flat events. Hence, the performance scores of the nested events inevitably include the performance on flat events.

### Evaluation settings

We evaluate the two event detection models (EXNN and SBNN) in two ways, (i) Against the event detection component of a state-of-the-art event extraction model (TEES), namely, *Event Detection Comparison*, (ii) As an event detection component within a pipeline model, with state-of-the-art Named Entity and Relation Detection components, namely, *End-to-end Event Extraction*. For SBNN, we choose k=8 for the experiments, as it achieves the best performance [25].

#### Event detection comparison

TEES [15] models event extraction as a series of classification tasks with the dependence on syntactic and dependency path features, which they acquire with the use of external tools. In contrast, we use neither syntactic nor any other external features, relying only on the data provided by the task. For our experiments we compare with TEES *single* models in contrast to the ensemble methods, as this enables us to make a direct comparison with TEES in a minimal setting. We evaluated the TEES published trained models on the tasks while keeping the same train/dev/test splits for our models. Our models were trained using the predicted relations from TEES previous components merged with the pairwise relations decomposed from the gold events. During inference, we predict event structures using only the predicted relations from TEES. We exclude feature- or rule-based models in our comparison, since they reported lower performance in our initial experiments (specifically the EventMine system [33]) when compared to the neural-based model TEES. We evaluate both models, EXNN and SBNN against TEES on the three BioNLP Shared Task 2013 datasets: Cancer Genetics (CG), Pathway Curation (PC) and GENIA Event Extraction for NFkB knowledge base (GE).

#### End-to-end event extraction

To evaluate the upper-bound and lower-bound performance of our proposed models, we performed experiments using neural-based state-of-the-art components for named entity and relation detection into a pipeline approach. In the pipeline approach as proposed in [15, 24], the output of each component serves as the input for the next component. For example, the event structure detection component takes as input both the output of the relation extraction component and the entity and trigger detection components.

We set up three different pipeline training scenarios to evaluate the event detection model. Each scenario uses different inputs to train the event detection model and predict events. In scenario 1 (upper-bound), we use gold relations and entities to train the event detection model and predict event structures. This will set the upper-bound recall of our proposed models. In scenario 2 (pipeline), we train each component using gold relations and entities but predict events using predicted relations and entities. Following TEES, correctly predicted relations along with false positives are included into the training data to enable the model to handle noise during inference. This scenario will measure the performance of our event detection model against the state-of-the-art, which we described in the previous section. In scenario 3 (lower-bound), we train each component using the predictions from the previous models in the pipeline. More specifically, the relation extraction component uses the predictions of named entity and trigger detection module, and the event detection model uses predicted entities, triggers and relations during training. As expected, this will result in lowest performance for the event model thus setting its lower-bound.

We used the models of Ju et al. [3] for named entity and trigger detection and Christopoulou et al. [34] for relation extraction, respectively. In the following paragraphs we describe how we incorporate each of these components into the event detection pipeline. To extract nested named entities and triggers, we applied the layered BiLSTM-CRF model [3]. In addition to the nestedness between either entities or triggers spans, entities can be also nested within triggers spans. Based on the observation that triggers depend on entities, we force the model to detect entities first, which are further used to encourage the detection of triggers. These predictions will be fed into the second component for relation extraction. We modified the CRF layer in the module following Minkov et al. [35] to increase the recall of entities and triggers and alleviate error propagation to the next component. Specifically, we first tuned the layered BiLSTM-CRF using Bayesian Optimisation [36] on the development set to get the best model. Then, we applied extractor tweaking [35] to the CRF layer, producing higher recall without significantly hurting the recall-precision trade-off.

Regarding the extraction of relations for event detection, we modified the relation extraction model proposed by Christopoulou et al. [34]. The first step involved breaking down all events into binary interactions, using the argument roles as semantic relations between trigger-argument pairs. In particular, we enable Trigger-Entity and Trigger-Trigger associations, forcing Entity-Trigger pairs to share the “no relation” category. In case the models were trained using gold annotated data (*scenario 1*), we augmented the training dataset with additional pairs, using equivalent entities that exist in the training set (marked with $^*$Equiv in the original annotation files). In case an argument role contained enumeration (e.g. Theme1), this was removed and the role without enumeration was used instead (e.g. Theme). In case where models were trained using predicted training data (*scenario 2*), we merged the predictions of the Named Entity Recognition module with the gold annotations by keeping only the correctly identified (True Positives) as well as the incorrectly identified (False Positives) entities and triggers. We enabled the usage of different embedding spaces to embed relative position embeddings to the first and the second argument of each pair, respectively. Finally, we allowed relations between nested named entities and triggers.

We perform our experiments of the pipeline models on the two bigger datasets, the BioNLP 2013 Cancer Genetics (CG) and the Pathway Curation (PC) dataset.

### Quantitative results

#### Event detection results

Table 3 shows the event detection performance of the proposed models on all events of the BioNLP 2013 shared tasks test sets compared to the TEES system [15].

**Table 3:** Event detection performance on the BioNLP 2013 shared tasks test sets

Dataset	Model	P	R	F1
CG 2013	TEES	0.6142	**0.5293**	0.5686
	EXNN	**0.6555**	0.4810	0.5549
	SBNN	0.6367	0.5143	**0.5690**
PC 2013	TEES	0.5885	0.4790	**0.5281**
	EXNN	**0.6151**	0.4088	0.4912
	SBNN	0.5531	**0.4855**	0.5171
GE 2013	TEES	0.5895	**0.4029**	**0.4787**
	EXNN	0.5925	0.3881	0.4690
	SBNN	**0.6155**	0.3859	0.4744

Bold indicates best performing Precision (P), Recall (R) and F1 measure for the respective scenarios and models

Both the EXNN and SBNN models yield higher precision in two datasets (CG and GE, respectively). This can be attributed to the bottom-up prediction mechanism which only predicts nested events once flat events are predicted. This leads to less noise and more precision, which in turn also affects recall. In the CG dataset, which is the biggest dataset in terms of event instances and proportion of nested and overlapping events, SBNN outperforms all the other models. Using the Approximate Randomisation test [37], we validated that there is no significant statistical difference between SBNN and TEES F1-score performance (significance at $p < 0.05$). Thus, we conclude, that the SBNN model achieves performance comparable to the TEES event detection module without using syntactic features or external tools. This finding suggests that the SBNN model can be applied to other domains with no need for feature engineering.

#### End-to-end event extraction results

Tables 4 and 5 show the performance of the event detection models for the three scenarios: upper-bound, pipeline, lower-bound on the development sets of BioNLP 2013 CG and PC respectively.

**Table 4:** Performance comparison between for three pipeline scenarios on the CG 2013 development set

Scenario	Model	P	R	F1
Upper-bound	EXNN	**0.9762**	0.8745	0.9225
	SBNN	0.9700	**0.8979**	**0.9326**
Pipeline	EXNN	0.5690	0.5331	0.5505
	SBNN	**0.5926**	**0.5384**	**0.5642**
Lower-bound	EXNN	0.5621	0.3332	0.4184
	SBNN	**0.5656**	**0.3403**	**0.4249**

Bold indicates best performing Precision (P), Recall (R) and F1 measure for the respective scenarios and models

**Table 5:** Performance comparison between for three pipeline scenarios on the PC 2013 development set

Scenario	Model	P	R	F1
Upper-bound	EXNN	**0.9528**	0.8029	0.8715
	SBNN	0.9431	**0.8366**	**0.8867**
Pipeline	EXNN	0.5167	0.4598	0.4866
	SBNN	**0.5322**	**0.4796**	**0.5045**
Lower-bound	EXNN	0.4678	0.2435	0.3203
	SBNN	**0.4748**	**0.2642**	**0.3395**

Bold indicates best performing Precision (P), Recall (R) and F1 measure for the respective scenarios and models

For the pipeline scenario, where the models are trained on gold relations and evaluated on predicted relations, the SBNN model outperforms EXNN on both sets. These predicted relations were extracted using the relation extraction system of Christopoulou [34] trained on gold entities. We can see that the scores are lower than in upper-bound scenario where event detection models rely on gold relations.

The lower-bound scenario shows the performance of the event detection models when the pipeline components are trained on the predictions of previous components. This scenario results in the lowest scores, setting the lower-bound performance on the event detection component as expected. Between the models, the search-based model yields a higher F1-score.

In the three pipeline scenarios, the results show that the SBNN model performs consistently better than the EXNN model and thus corroborates the reported performance against the state-of-the-art in the previous section.

## Discussion

In this section, we discuss the different aspects of the SBNN model and perform error analysis. We focus our analysis on BioNLP CG 2013 development dataset.

### Model analysis

The performance of our model on the test sets (Table 3) showed that we can achieve comparable performance with the state-of-the-art TEES model but without the syntactic features or the external tools that the latter leverages. This suggests that our model is easier to apply to other domains. Another observation from the results is the relatively low F1-scores on the GE dataset for both TEES and our model. This can be attributed to GE’s high number of inter-sentence events ($22.55 \%$ vs $2.41 \%$ vs $3.11 \%$) and percentage of unknown words ($16.84 \%$ vs $11.66 \%$ vs $10.63 \%$), shown in Table 2, compared to PC and CG datasets respectively. These inter-sentence events cannot be filtered since we do not have access to the test sets as stated previously.

**Table 6:** Performance comparison on nested and overlapping event detection on the CG task 2013 development set

Model	Nested	Overlap	Flat
TEES	0.4270	0.3449	0.5681
EXNN	**0.4714**	**0.3785**	**0.6190**
SBNN	0.4524	0.3692	0.6050

Bold indicates best performing Precision (P), Recall (R) and F1 measure for the respective scenarios and models

Table 6 shows the performance of the models on nested and overlapping events in terms of F1 score, on the CG task 2013 development set. The results were obtained by taking into account the whole DAG structure of the predicted and gold events. Results show that both SBNN and EXNN outperformed TEES, confirming that our neural-based models can efficiently capture nested and overlapping event structures better. The EXNN model performs slightly better than SBNN as expected since it is an exhaustive method at the cost of more computation and with a lower performance in comparison with SBNN.

We also observe that the performance of our pipeline model which uses predicted entities (Table 4) is better than TEES which uses gold entities (Table 6), with 0.5505 (EXNN) and 0.5642 (SBNN) vs 0.5216 (TEES) F1-score, on the CG 2013 development set. Furthermore, our pipeline model (either using EXNN or SBNN) performs better than the pipeline model of DeepEventMine [20] (Table 3 in their paper) with F1-score of 0.5020 using predicted entities, despite the usage of BERT embeddings in their input representation. This finding would make our pipeline model the state-of-the-art model among pipeline systems. However, we leave further comparisons as part of future work.

### Error analysis

We perform error analysis of SBNN model on the BioNLP CG 2013 development set (see Additional file 3). Particularly, we focus our analysis on those event types with F1-scores lower than $50 \%$, which attribute to 17 out of 40 event types. Out of these, seven (7) have F1-scores equal to zero, which are due to the data sparsity in the training and development sets ($<5$ and $<15$ instances in train and development sets respectively, except *DNA methylation*), hence their low performance.

Since our model is trained to predict events in a bottom-up manner, we also observed a reasonable difficulty in predicting deeply nested events. Concretely, the results showed that out of the 17 event types with F1-scores less than $50 \%$, four (4) event types have the largest number of training instances (at least 200). These include: *Regulation*, *Positive Regulation*, *Negative Regulation* and *Planned Process*. In our analysis, we found that these event types are the most frequent having at least two (2) arguments, indicating their complexity given the search-based prediction process. This is relative to most of the other event types which only have one argument. Another finding is that the three regulation-related events have the largest depth of nested structures. Although our model outperforms the TEES model in predicting nested and overlapping events, we can use this finding to further investigate how to improve the performance.

While SBNN has higher precision, TEES has higher recall. This is expected since the latter generates all possible candidate events from predicted relations [15] and classifies them. The higher precision of our model, especially in the nested and overlapping structures such as *Regulation* (39.49 vs 32.47), *Negative Regulation* (54.34 vs 51.27), *Positive Regulation* (52.25 vs 45.40) and *Planned Process* (54.85 vs 51.83), can be attributed to the bottom-up search prediction procedure.

We also plot the distribution of event types with a particular number of arguments since our search-based model applies actions to each argument at every time step. The more arguments there are, the more time steps the search-based model would need to learn an event structure.

**Fig. 6:**
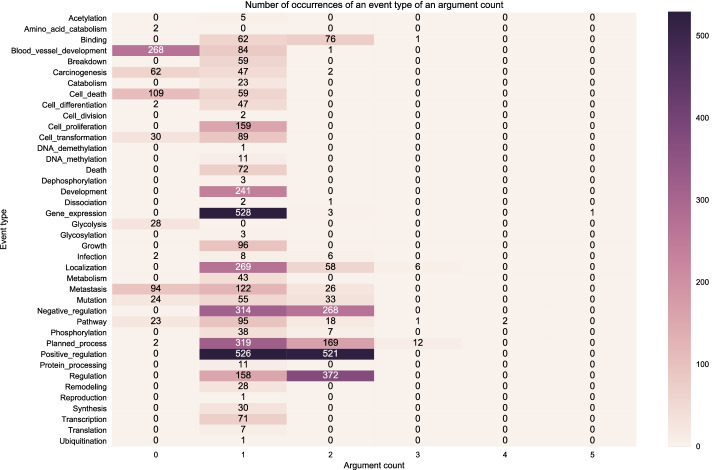
Distribution of event structures according to number of arguments in the BioNLP CG 2013 gold training set (gold relations)

Figure 6 shows a heatmap indicating the number of event type instances in the gold training set with a particular number of arguments. Darker shades mean that it has a relatively high number of instances which is indicated by the number. For example, Fig. 6 shows that *Positive Regulation* event type has 1,047 instances which can be broken down into the following: zero (0) instances with zero (0) argument, 526 instances having one argument and 521 instances having 2 arguments. We can observe that most of the event types have at least one argument. Furthermore, there are specific event types that have both one and two arguments such *Positive Regulation, Negative Regulation, Regulation*. Some event types mostly appear as one-argument events such as *Gene Expression, Growth, Cell Proliferation, Cell Transformation, etc*. Figure 6 also shows an outlier event structure of type *Gene Expression* which has one occurrence with 5 arguments.

**Fig. 7:**
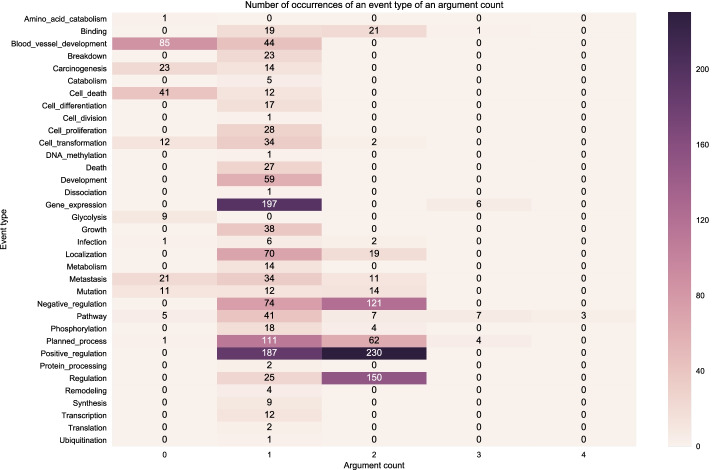
Distribution of event structures according to number of arguments in the BioNLP CG 2013 gold development set (gold relations)

Figure 7 shows the distribution of event structure instances on the development set using the gold relations. We can observe the training (Fig. 6) and development set (Fig. 7) have a relatively similar distribution of event types and the occurrences of event structures with a particular number of arguments. However, we can clearly see that the event type distribution is imbalanced in both data partitions.

**Fig. 8:**
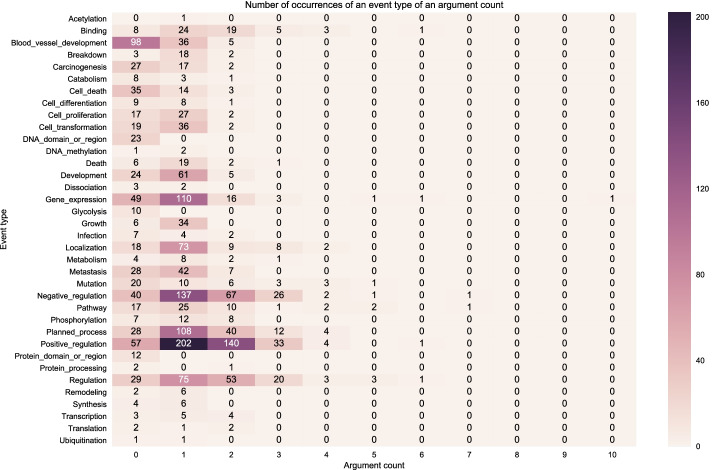
Distribution of event structures according to number of arguments in the BioNLP CG 2013 predicted development set (predicted relations)

Figure 8 shows the distribution of event structures using the predicted relations on the development set. We can observe that compared to the event structures from the gold relations in Fig. 7 the event structures distribution in Fig. 8 follows the same pattern in terms of the distribution of event types occurrences but in a slightly more diffuse manner, that is, over many argument counts. For example, while in Fig. 7 the event structure instances of *Positive Regulation* appear only as having one or two arguments, in Fig. 8*Positive Regulation* has instances with zero (0) to six (6) arguments though most of them are still in the 1-2 arguments, which is expected. The reason for this deviation from the gold relations is that the predictions contain false positive relations.

**Fig. 9:**
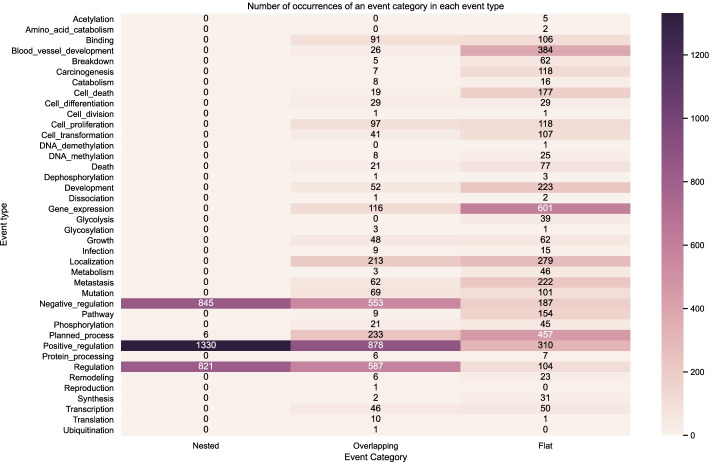
Distribution of event structures according to the number of nested, overlapping and flat events in the BioNLP CG 2013 gold training set (gold relations)

**Fig. 10:**
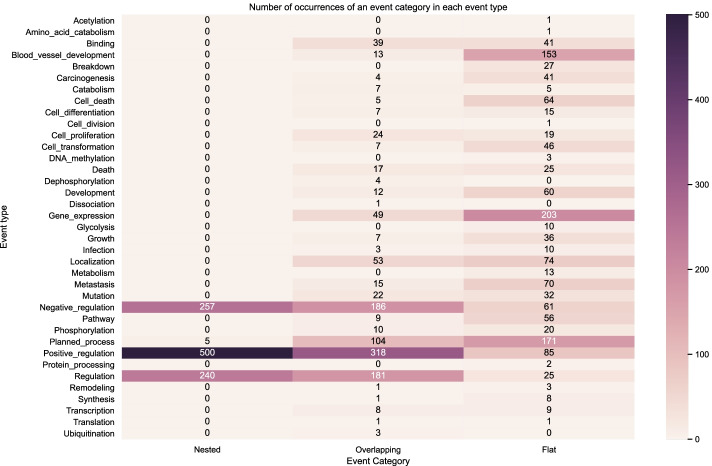
Distribution of event structures according to the number of nested, overlapping and flat events in the BioNLP CG 2013 gold development set (gold relations)

**Fig. 11:**
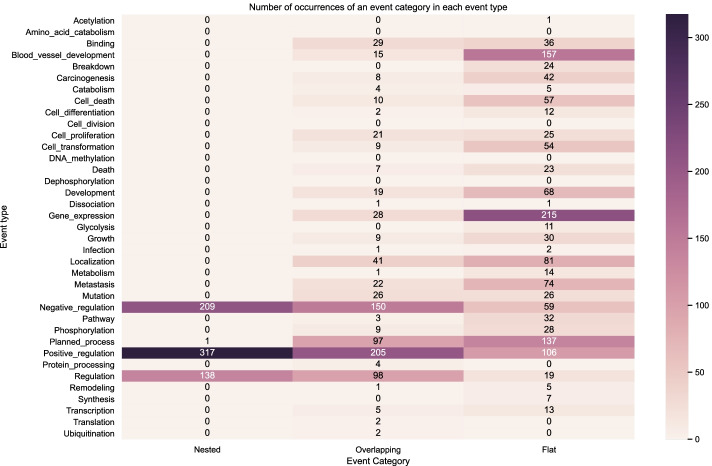
Distribution of event structures according to the number of nested, overlapping and flat events in the BioNLP CG 2013 predicted development set (predicted relations)

Figure 9 shows the distribution of the event structures according to the number of nested, overlapping and flat events in the BioNLP CG 2013 gold training set. Figures 10 and 11 show the distribution on the development set using gold relations and predicted relations respectively. We can observe that visually our predictions match closely the distribution across event types of the training and development gold sets, indicating that our model learned to capture the nested and overlapping events. More specifically, we notice that the event types in descending order of counts for nested events in both the training and development sets are the following: *Positive regulation*, *Negative regulation* and *Regulation*. This same sequence is true for the predictions of our model. Our model also predicts the most overlapping events in the *Positive regulation* and the most flat in *Gene expression* which correspond to the types that have the most occurrences in each category for the training and development set.

The uneven distribution of event categories across event types showed visually via the heatmaps highlights the challenge that models need to capture. For example, in both the training and development set we have occurrences for *Amino acid catabolism* but this is never predicted by our models and this can be attributed to the very low number of occurrences in the said dataset partitions. However, in some event types such as *Ubiquitination*, our model is able to predict correctly despite the low number of occurrences. In this case, it may be that the surrounding semantic context provides enough information to disambiguate such cases.

#### Computation efficiency

To compare the computational efficiency, we counted the number of classifications (or action scoring functions calls) performed by each model on Cancer Genetics 2013 development set. We choose the number of classifications as a computational efficiency metric since it is independent of the computer architecture. The computational efficiency of SBNN depends on the parameter *k* that defines the beam search width, and for the comparison we choose the best performing value based on the event detection performance ($k=8$).

**Table 7:** Computation efficiency on the CG 2013 development set

Model	Number of Classifications
TEES	6141
EXNN	25,766
SBNN	**4093**

Bold indicates best performing Precision (P), Recall (R) and F1 measure for the respective scenarios and models

Table 7 shows the number of classifications performed by the different models on the CG 2013 development set. SBNN requires about two-thirds the computational cost than TEES and 6 times less than EXNN. SBNN performs fewer classifications because of its threshold and beam width k parameters, which filter and effectively limit the event structures that will be passed to the neural network for classification. Therefore, SBNN is more computationally efficient than TEES and EXNN.

## Conclusion

In this work, we compared two neural models for nested and overlapping event detection: a novel EXhaustive Neural Network model and a Search-Based neural Network model. The SBNN model outperforms the EXNN model and achieves comparable performance with the state-of-the-art TEES event detection model without using syntactic features or external tools. Experimental analyses revealed some desirable characteristics of the SBNN model, such as its flexibility and computational efficiency, while EXNN was found to be better in capturing nested and overlapping event structures.

As future work, we aim to apply the models to other DAG structures such as nested/discontinuous entities [38]. Furthermore, we will investigate contextualised input representations (e.g. BERT embeddings [39]) to improve the event detection especially for sparse event types and deeply nested structures. Finally, a more detailed comparison between our models and DeepEventMine [20] in the pipeline setting is needed to confirm our previous observations.

## Supplementary information

**Additional file 1.** A schematic architecture of the search-based model (SBNN) detecting structures in a bottom-up manner.

**Additional file 2.** More information on the scoring function of the search-based model (SBNN).

**Additional file 3.** Detailed performance comparison of SBNN and TEES on CG 2013 data set.

## Data Availability

The BioNLP 2013 Shared Tasks, including Cancer Genetics and Pathway Curation is available at http://2013.bionlp-st.org/tasks.

## Abbreviations

SBNN: Search-based neural network
EXNN: Exhaustive neural network
TEES: Turku event extraction system
CG: Cancer genetics
PC: Pathway curation
GE: Genia
DAG: Directed acyclic graph
LSTM: Long short-term memory
BiLSTM: Bidirectional long short term memory
CRF: Conditional random field

## Footnotes

1 More details can be found in the shared task website: http://2013.bionlp-st.org/tasks/cancer-genetics.
