# Supplementary figures and images for: Comparing neural models for nested and overlapping biomedical event detection
Source: BMC Bioinformatics. 2022 Jun 2;23:211. doi: 10.1186/s12859-022-04746-3 (PMC9161617; doi:10.1186/s12859-022-04746-3)

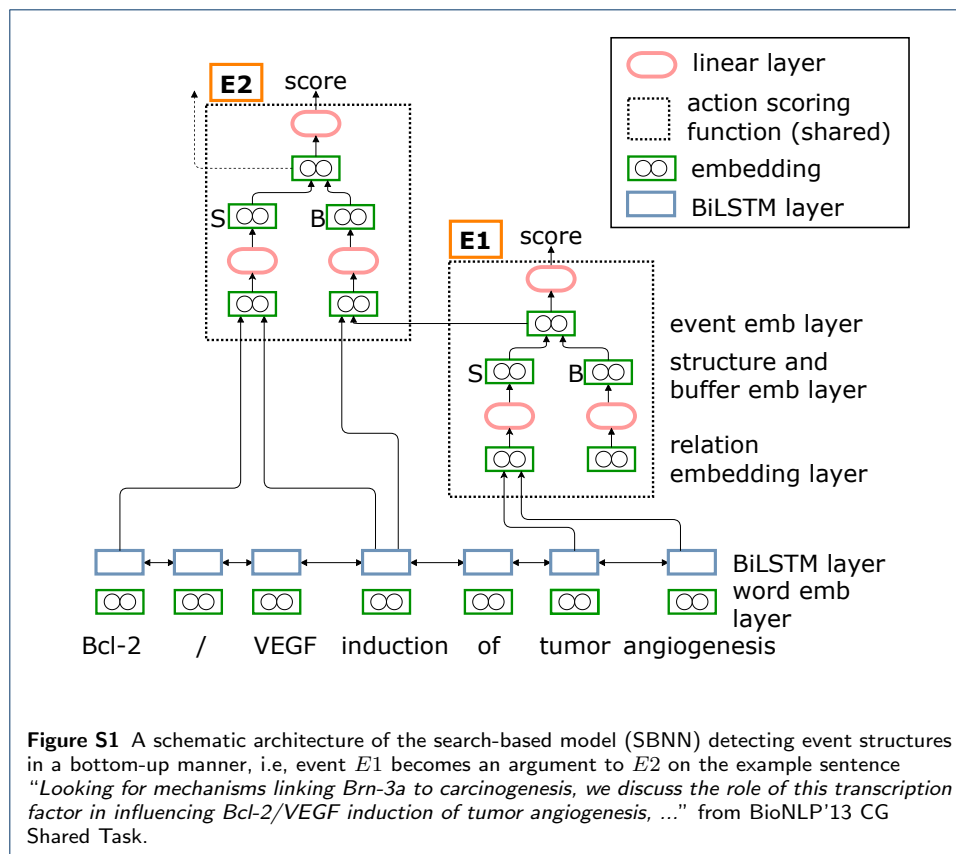

Supplement: Supplementary file 1 — Additional file 1. A schematic architecture of the search-based model (SBNN) detecting structures in a bottom-up manner. [file 12859_2022_4746_MOESM1_ESM.pdf]
