# Supplementary material for: Comparing neural models for nested and overlapping biomedical event detection
Source: BMC Bioinformatics. 2022 Jun 2;23:211. doi: 10.1186/s12859-022-04746-3 (PMC9161617; doi:10.1186/s12859-022-04746-3)
Supplement: Supplementary file 2 — Additional file 2. More information on the scoring function of the search-based model (SBNN). [file 12859_2022_4746_MOESM2_ESM.pdf]

Scoring Function

We employ a **BiLSTM** network to generate the word representations from pre-trained **word embeddings**. To represent phrases, we averaged the word representations. The LSTM network is shared among the states during search in the sentence. We build a relation embedding for each argument, which concatenates the information of the trigger t, the role o, the argument a and the action c. We include both the type information p and the word or phrase representation w of the trigger or entity argument. Formally, each relation r_i is represented as a **relation embedding**: $\mathbf{r}_i = [\mathbf{t}_p; \mathbf{t}_w; \mathbf{o}_p; \mathbf{a}_p; \mathbf{a}_w; \mathbf{c}]$, where $\mathbf{t}_p$ is the representation of the type of the trigger and so on. Each $\mathbf{r}_i$ is passed to a linear **hidden layer**, then to a rectified linear unit (ReLU) non-linearity and summed to produce the **structure** and **buffer** embeddings: $\mathbf{S}_t$ and $\mathbf{B}_t$.

We use a neural network as the **action scoring function** (indicated by the dotted box in Additional file 1) defined as $\sigma(a_t|S_{t-1}, B_{t-1})$. We model the action scoring function using S_t and B_t, which are composed by adding an action a_t for a relation r_t to S_{t-1} and moving r_t from B_{t-1} to S_{t-1}. The state at any time step t is composed of the buffer B_t and the partially built structure S_t. Each of S_t and B_t contains a set of relations $\{r_1, r_2, r_3, \ldots, r_n\}$. In Additional file 1, there is no arrow to B in event *E1* because the diagram only shows the model snapshot at a specific time step during the search process. In this particular time step t, the buffer B in *E1* is already empty and thus, it does not contain any relations $\mathbf{r}_i$.

$\mathbf{S}_t$ and $\mathbf{B}_t$ are then concatenated to form the **event embedding**. The event embedding has the same dimension as the sum of argument type and word dimensions so that it can be used as argument representation in nested events as shown in Additional file 1. Then, we passed the event embedding into a linear **hidden layer** and output z_t. Finally, the scoring function σ is calculated as $\sigma(a_t|S_{t-1}, B_{t-1}) = \text{sigmoid}(z_t)$.
