# Supplementary material for: Comparing neural models for nested and overlapping biomedical event detection
Source: BMC Bioinformatics. 2022 Jun 2;23:211. doi: 10.1186/s12859-022-04746-3 (PMC9161617; doi:10.1186/s12859-022-04746-3)
Supplement: Supplementary file 3 — Additional file 3. Detailed performance comparison of SBNN and TEES on CG 2013 data set. [file 12859_2022_4746_MOESM3_ESM.pdf]

Table A1 Detailed performance of the SBNN and the TEES models on the development set of the CG 2013 data set.

EVENT CLASS	SBNN (%)			TEES (%)		
	P	R	F1	P	R	F1
Development	73.49	83.56	78.21	73.26	85.14	78.75
Blood_vessel_developm	85.35	85.35	85.35	80.46	84.34	82.35
Growth	97.44	88.37	92.68	97.44	88.37	92.68
Death	64.52	48.78	55.56	61.76	50.00	55.26
Cell_death	76.12	73.91	75.00	76.47	75.36	75.91
Cell_proliferation	82.61	86.36	84.44	84.44	86.36	85.39
Cell_division	0.00	0.00	0.00	0.00	0.00	0.00
Cell_differentiation	84.62	45.83	59.46	85.71	50.00	63.16
Remodeling	50.00	75.00	60.00	60.00	75.00	66.67
Reproduction	100.00	100.00	100.00	100.00	100.00	100.00
=[ANATOMY-TOTAL]=	80.54	78.07	79.29	78.92	78.59	78.76
Mutation	0.00	0.00	0.00	25.37	28.81	26.98
Carcinogenesis	66.67	69.57	68.09	67.35	71.74	69.47
Cell_transformation	71.43	80.36	75.63	71.43	80.36	75.63
Breakdown	64.00	69.57	66.67	65.38	62.96	64.15
Metastasis	70.00	73.26	71.59	69.57	74.42	71.91
Infection	66.67	46.15	54.55	50.00	53.85	51.85
=[PATHOL-TOTAL]=	68.94	57.24	62.55	58.84	63.76	61.20
Metabolism	66.67	71.43	68.97	66.67	71.43	68.97
Synthesis	85.71	66.67	75.00	85.71	60.00	70.59
Catabolism	44.44	36.36	40.00	57.14	30.77	40.00
Glycolysis	54.55	60.00	57.14	54.55	60.00	57.14
Amino_acid_catabolism	0.00	0.00	0.00	0.00	0.00	0.00
Gene_expression	85.40	71.48	77.82	83.76	70.25	76.41
Transcription	69.23	52.94	60.00	58.82	55.56	57.14
Translation	0.00	0.00	0.00	0.00	0.00	0.00
Protein_processing	50.00	50.00	50.00	0.00	0.00	0.00
Acetylation	0.00	0.00	0.00	0.00	0.00	0.00
Glycosylation	100.00	100.00	100.00	100.00	100.00	100.00
Phosphorylation	72.97	81.82	77.14	73.68	84.85	78.87
Ubiquitination	66.67	66.67	66.67	0.00	0.00	0.00
Dephosphorylation	0.00	0.00	0.00	0.00	0.00	0.00
DNA_methylation	50.00	33.33	40.00	100.00	100.00	100.00
DNA_demethylation	100.00	100.00	100.00	100.00	100.00	100.00
Pathway	45.71	23.19	30.77	38.46	25.32	30.53
=[MOLECUL-TOTAL]=	75.62	61.20	67.65	72.19	59.70	65.36
Binding	70.59	12.90	21.82	52.54	31.96	39.74
Dissociation	0.00	0.00	0.00	0.00	0.00	0.00
Localization	65.22	44.12	52.63	45.45	50.00	47.62
=[GENERAL-TOTAL]=	64.86	31.44	42.35	47.20	42.44	44.69
Regulation	39.49	21.23	27.62	32.47	21.93	26.18
Positive_regulation	52.25	34.37	41.46	45.40	33.61	38.62
Negative_regulation	54.34	44.24	48.77	51.27	47.03	49.06
=[REG-TOTAL]=	50.73	34.32	40.94	44.60	34.43	38.86
Planned_process	54.85	40.50	46.60	51.83	39.37	44.75
===[SUB-TOTAL]===	63.60	47.46	54.36	56.81	48.21	52.16
Negation	0.00	0.00	0.00	42.17	29.66	34.83
Speculation	0.00	0.00	0.00	27.84	23.28	25.35
==[MOD-TOTAL]==	0.00	0.00	0.00	34.44	26.50	29.95
====[TOTAL]====	63.60	44.09	52.07	55.43	46.74	50.72
